# Association Between Insulin Resistance Markers and Immunological Markers in Gestational Diabetes Mellitus

**DOI:** 10.7759/cureus.106164

**Published:** 2026-03-30

**Authors:** Vandana Tiwary, Monisha M, Ramesh R, Bhabani Pegu

**Affiliations:** 1 Biochemistry, Jawaharlal Institute of Postgraduate Medical Education and Research, Puducherry, IND; 2 Obstetrics and Gynecology, Jawaharlal Institute of Postgraduate Medical Education and Research, Puducherry, IND

**Keywords:** adipokines, chemerin, c-peptide, gestational diabetes mellitus, insulin resistance, interleukin-6

## Abstract

Introduction

Gestational diabetes mellitus (GDM) is characterized by insulin resistance with inadequate β-cell compensation, resulting in maternal hyperglycemia and adverse fetal outcomes. Emerging evidence highlights the role of inflammatory cytokines and adipokines in driving the underlying immunometabolic changes associated with this condition.

Objective

This study aimed to investigate the interplay of inflammatory cytokines (IL-6, IL-10, and IL-13), adipokines (chemerin and visfatin), and C-peptide in women with GDM among the South Indian population.

Methods

This case-control study included 88 pregnant women (44 with GDM and 44 normoglycemic controls). Fasting plasma glucose, C-peptide, interleukins (IL-6, IL-13, and IL-10), and adipokine markers chemerin and visfatin were measured. Associations were explored using Spearman’s correlation analysis. Multiple linear regression was performed as the primary analysis with fasting glucose as the dependent variable to identify independent immunometabolic determinants of glycemia. Binary logistic regression was conducted as a secondary analysis to identify glucose-independent predictors of GDM status. Results were expressed as regression coefficients and odds ratios (OR) with 95% confidence intervals (CI). Data were analyzed using IBM SPSS Statistics for Windows version 19.0 (IBM Corp., Armonk, NY).

Results

Women with GDM had significantly higher fasting glucose and lower C-peptide levels compared to controls (p < 0.05). Fasting glucose showed an inverse correlation with IL-6 and C-peptide, and a positive correlation with chemerin. Visfatin showed a positive correlation with IL-13. In multiple linear regression, IL-6 and C-peptide were independently and negatively associated with fasting glucose, while chemerin and gestational age were positive predictors (adjusted R² = 0.201; p < 0.001). In logistic regression, higher chemerin levels were associated with increased odds of GDM (OR = 1.010; 95% CI: 1.005, 1.016), whereas higher C-peptide (OR = 0.672; 95% CI: 0.536, 0.843) and IL-6 levels (OR = 0.796; 95% CI: 0.712, 0.890) were associated with reduced odds of GDM (all p < 0.01).

Conclusion

Immunological marker IL-6 and adipokine marker chemerin are independently associated with glycemic status. Chemerin may appear to contribute to insulin resistance, while IL-6 and C-peptide may reflect compensatory immune and β-cell responses during pregnancy. These findings suggest the complex immune-metabolic interplay underlying gestational diabetes mellitus.

## Introduction

The most common metabolic disorder in pregnancy is gestational diabetes mellitus (GDM), which is known to result in significant adverse maternal and neonatal complications [1-3]. Chronic insulin resistance is the key metabolic disturbance in gestational diabetes mellitus (GDM). During normal pregnancy, insulin resistance increases physiologically to ensure an adequate glucose supply to the growing fetus. As pregnancy progresses, insulin demand rises, and the combination of pancreatic β-cell insufficiency may lead to impaired glucose tolerance and ultimately to GDM [4].

In addition to metabolic alterations, chronic low-grade inflammation and immune system alterations may also play important roles in the pathogenesis of GDM. An exaggerated proinflammatory state can disrupt the insulin signalling pathway, worsening insulin resistance [5,6]. Also, adipose tissue plays a pivotal role in linking inflammation and insulin resistance by secreting adipokines and other immunometabolic regulators [7].

The national prevalence of GDM in India is around 22.4%, indicating that nearly one in four pregnant women is affected, with considerable regional variation [8]. This substantial burden underscores the need to explore insulin resistance and immunological markers, and their associations, to strengthen screening and diagnostic strategies for GDM.

The most commonly used biochemical markers for endogenous insulin production and insulin resistance are fasting insulin and C-peptide. In GDM, due to peripheral insulin resistance, circulating insulin and C-peptide levels increase, leading to compensatory hyperinsulinemia [4]. Among the inflammatory mediators, IL-6 is a pleiotropic cytokine with proinflammatory and anti-inflammatory properties and plays a vital role in insulin resistance by impairing insulin receptor signalling and enhancing hepatic gluconeogenesis, whereas IL-10 and IL-13 are anti-inflammatory cytokines that act in opposition to IL-6. However, the detailed mechanism underlying their role remains to be elucidated [9-11].

Visfatin, an adipokine that is produced by visceral adipose tissue, has insulin-like effects. Its level is elevated in obesity and GDM, implying an important role in the pathophysiology of the disease [12,13]. Chemerin, another adipokine also known as tazarotene-induced gene 2 (TIG2) or retinoic acid receptor responder 2 (RARRES2), secreted mainly by white adipose tissue, impairs glucose tolerance and increases insulin resistance during pregnancy by activating adipocyte differentiation and immune cell recruitment, found to be elevated in GDM [14].

Many studies have examined the role of inflammatory mediators in GDM, but few have examined markers of insulin resistance and inflammatory mediators together [15]. In our previous study using the same study group, we observed increased chemerin levels along with decreased IL-6 levels in women with GDM. Serum IL-6 levels were lower in the GDM group compared to the non-GDM group (4.41 pg/mL {2.84-8.52} versus 24.58 pg/mL {14.94-59.96}, p < 0.001). Chemerin levels were higher in the GDM group than in the non-GDM group (228.66 ng/L {205.71-284.31} versus 180.87 ng/L {151.12-235.80}, p = 0.003). Building upon these findings, the present study aims to evaluate the association between markers of insulin resistance (C-peptide, chemerin, and visfatin) and immunological markers (IL-6, IL-10, and IL-13) within this cohort. A combined evaluation of markers of insulin resistance and immunological markers may lead to a better understanding of the disease mechanism underlying GDM and help identify biomarkers and novel therapeutic targets.

## Materials and methods

Study design and ethical approval

**Figure 1:**
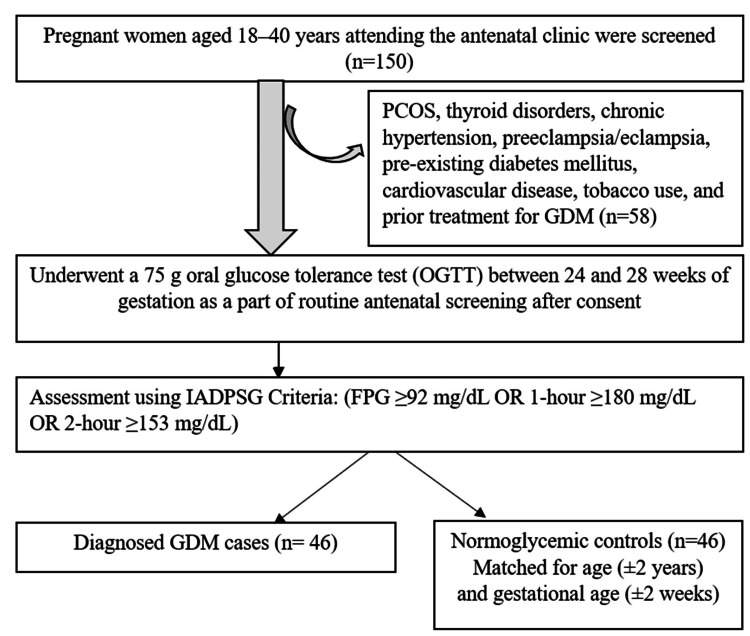
Flow of participant selection and recruitment n, number of participants; FPG, fasting plasma glucose; GDM, gestational diabetes mellitus; OR, odds ratio; PCOS, polycystic ovarian syndrome

This case-control study was conducted in the Departments of Biochemistry and Obstetrics and Gynecology at our institute from July 2022 to June 2024. The ethical approval for this study was granted by the Institutional Ethics Committee for Observational Studies of Jawaharlal Institute of Postgraduate Medical Education and Research (JIPMER) (approval number JIP/IEC/2022/0148, dated 02/08/2022). After getting written informed consent, the participants were recruited for the study. Figure 1 shows the screening and recruitment process of the participants included in the study based on the inclusion and exclusion criteria and diagnosis based on the IADPSG criteria [16].

Sample size calculation

The sample size was calculated using previously reported mean and standard deviation values of the study parameters, which were small. Due to heterogeneity across earlier studies, a conservative, moderate effect size (Cohen’s d = 0.6) was assumed. Considering an alpha of 0.05 and 80% power, the sample size required for the study was calculated to be 44 participants per group. To account for potential attrition, 46 participants were enrolled in each group. Sample size estimation was performed using the nMaster 2.0 software (Department of Biostatistics, Christian Medical College, Vellore, India), and consecutive sampling was adopted.

Sample processing and biochemical analysis

For routine oral glucose tolerance test (OGTT) screening, 2 mL of peripheral venous blood was collected into a sodium fluoride tube, and 2 mL of blood was collected into a plain red-top tube for the study. The samples collected in plain tubes were allowed to clot at room temperature for 20 minutes and centrifuged at 3000-4000 revolutions per minute (rpm) for 10 minutes. The plasma glucose samples were analyzed using the hexokinase method on the Beckman Coulter AU5800 analyzer (Beckman Coulter, Inc., Brea, CA). Serum samples were aliquoted and stored at -40°C until enzyme-linked immunosorbent assay (ELISA) analysis, according to the manufacturer’s recommendations. Serum levels of chemerin, visfatin, IL-6, IL-10, IL-13, and C-peptide were measured in duplicate using commercially available enzyme-linked immunosorbent assay (ELISA) kits according to the manufacturer’s protocols. Details regarding the detection ranges, sensitivity, and intra- and inter-assay coefficients of variation are provided in the supplementary material (Appendices). A total of 92 samples were collected; however, 88 samples were analyzed due to financial constraints. The excluded samples were randomly selected to minimize bias and were not excluded based on clinical characteristics. Baseline parameters of the excluded participants were comparable to those of the analyzed participants.

Statistical analysis

Normality was assessed using the Shapiro-Wilk test. Continuous variables were expressed as mean ± standard deviation or median with interquartile range, depending on the data distribution. Correlation analysis was performed using Spearman’s correlation test based on the data distribution. Multiple linear regression analysis was performed with fasting plasma glucose as the dependent variable. Predictor variables were selected based on correlation, sample size considerations, and biological plausibility to avoid overfitting. Secondary binary logistic regression analysis was conducted to identify glucose-independent predictors of GDM status. Chemerin, IL-6, and C-peptide were included as independent variables. Plasma glucose was excluded from the model because it is part of the diagnostic criteria for GDM and could introduce circularity. Data analysis for this study was performed using SPSS Statistics version 19 (IBM Corp., Armonk, NY).

## Results

Demographics

A total of 88 pregnant women, 44 GDM cases and 44 non-GDM age-matched controls, were included in data analysis. The mean age of GDM cases is 26.34 ± 3.47 years, and that of non-GDM controls is 27.52 ± 4.72 years. Thirty-nine (88.6%) out of 44 women with GDM were from urban areas, while 36 (81.8%) out of 44 non-GDM women were from rural areas. The fasting glucose and C-peptide levels of the study participants are summarized in Table 1.

**Table 1:** Baseline fasting glucose and C-peptide levels in cases and controls Values are expressed as median (interquartile range) *P < 0.05 is considered statistically significant

	Cases	Controls	Z	P
Fasting glucose	95 (92-104.75)	79.5 (72.25-86)	-6.58	<0.001*
C-peptide	4.14 (3.05-5.56)	5.44 (4.04-6.54)	-2.56	0.01*

Baseline differences in inflammatory (IL-6, IL-10, and IL-13) and adipokine markers (chemerin and visfatin) between the study groups showed significantly higher chemerin levels and lower IL-6 levels in cases, as previously reported. In the present analysis, we examined the relationships between these inflammatory markers and glycemic parameters using correlation and multivariable modelling approaches.

Correlation analysis of insulin resistance markers and immunological markers

Spearman’s correlation analysis revealed significant associations between fasting plasma glucose and gestational age, IL-6, chemerin, C-peptide, and maternal age. C-peptide showed significant correlations with IL-6, IL-13, and chemerin, while visfatin correlated positively with IL-13 (Table 2).

**Table 2:** Correlation between insulin resistance markers and immunological markers Correlation analysis was performed using Spearman’s rank correlation test. Significant results are presented in the table *P < 0.05 is considered statistically significant

Variable pair	r	P
Fasting glucose-gestational age	0.24	0.02*
Fasting glucose-C-peptide	-0.29	0.006*
Chemerin-C-peptide	0.27	0.01*
Chemerin-fasting glucose	0.22	0.03*
IL-6-fasting glucose	-0.48	<0.001*
IL-6-C-peptide	0.47	<0.001*
IL-13-C-peptide	0.21	0.04*
IL-13-visfatin	0.44	<0.001*

**Figure 2:**
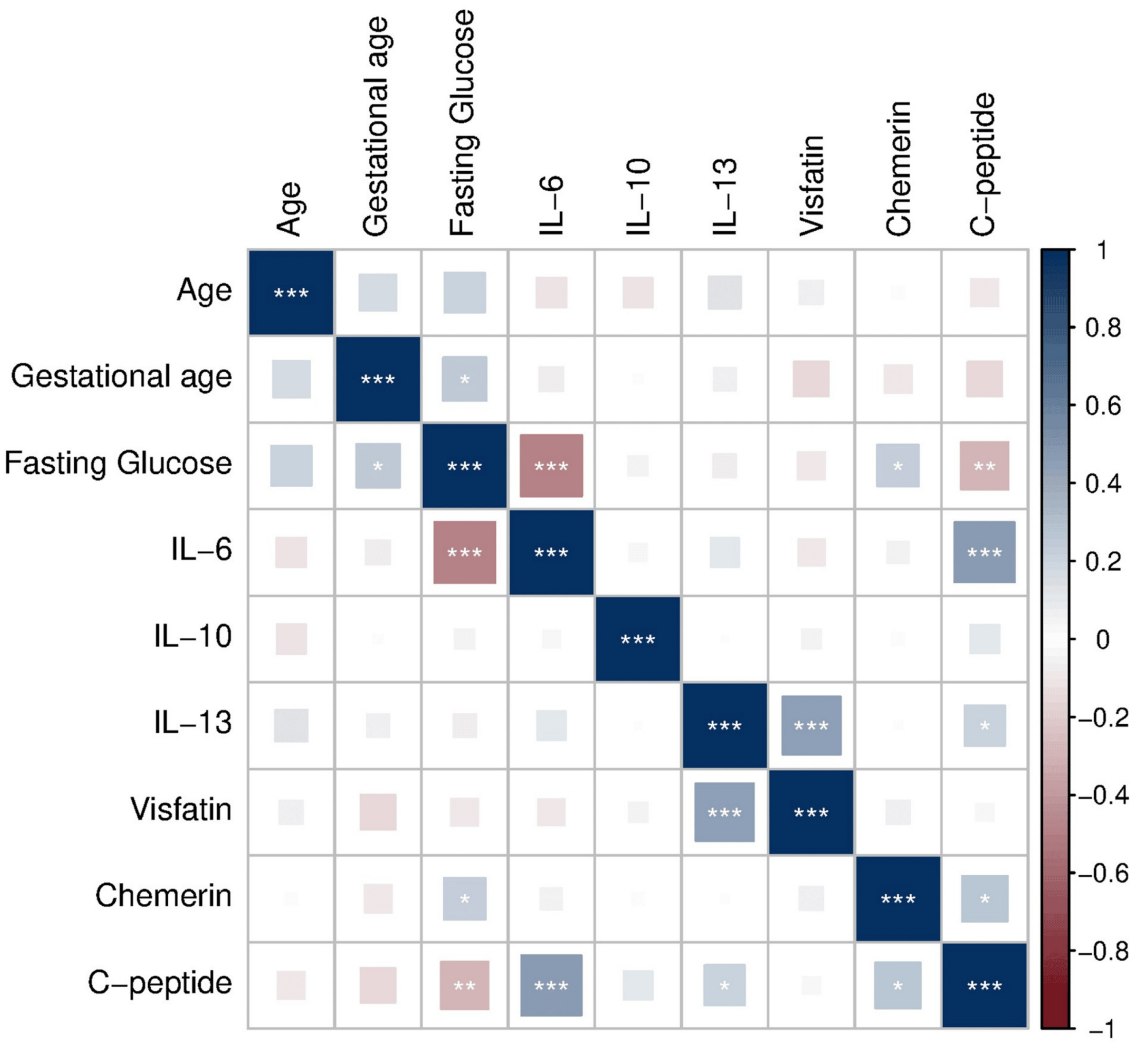
Heatmap illustrating associations between inflammatory markers, adipokines, and glycemic parameters The heatmap illustrates the correlation matrix between maternal age, gestational age, fasting glucose, inflammatory cytokines (IL-6, IL-10, and IL-13), adipokines (visfatin and chemerin), and C-peptide. Blue shades represent positive correlations, whereas red shades indicate negative correlations, with color intensity corresponding to the strength of the correlation coefficient. Statistically significant correlations are indicated by asterisks (*p < 0.05, **p < 0.01, and ***p < 0.001)

Figure 2 shows the correlation heatmap of relationships between gestational age, fasting glucose, C-peptide, adipokines, and inflammatory cytokines.

Multiple linear regression analysis

Table 3 shows multiple linear regression analysis with fasting glucose as the dependent variable, demonstrating that the overall model was statistically significant (R² = 0.238; adjusted R² = 0.201; p < 0.001). IL-6 and C-peptide were independently and negatively associated with fasting glucose, while chemerin and gestational age were independent positive predictors. These findings indicate that immunological and adipokine markers can independently be associated with glycemic status during pregnancy.

**Table 3:** Multiple linear regression analysis showing independent predictors of fasting glucose Multiple linear regression with fasting glucose as the dependent variable. R² = 0.238, adjusted R² = 0.201, F(4, 83) = 6.49, and p < 0.001 *P < 0.05 is considered statistically significant B, unstandardized coefficient; SE, standard error; β, standardized coefficient; t, t-statistic; 95% CI, 95% confidence interval

Predictor	B	SE	β	t	P	95% CI for B
Intercept	28.03	25.73	-	1.09	0.279	-23.14, 79.20
IL-6	-0.09	0.04	-0.24	-2.32	0.022*	-0.17, -0.01
C-peptide	-1.72	0.65	-0.80	-2.65	0.010*	-3.02, -0.43
Chemerin	0.05	0.02	0.91	3.09	0.003*	0.02, 0.08
Gestational age (weeks)	2.30	0.98	0.23	2.35	0.021*	0.36, 4.25

In secondary analysis, binary logistic regression (Table 4) identified chemerin, C-peptide, and IL-6 as independent predictors of GDM status. Higher chemerin levels were associated with increased odds of GDM. In contrast, higher C-peptide levels were associated with reduced odds of GDM. Similarly, IL-6 demonstrated an inverse association with GDM.

**Table 4:** Binary logistic regression analysis showing glucose-independent predictors of GDM Binary logistic regression identifying glucose-independent predictors of GDM. Values are OR with 95% CI. Note: outcome variable: GDM status (case and control) *P < 0.05 is considered statistically significant OR, odds ratio; CI, confidence interval; SE, standard error; GDM, gestational diabetes mellitus

Predictor	B	SE	Wald χ²	P value	OR	95% CI
Chemerin	0.010	0.003	12.56	<0.001*	1.010	1.005, 1.016
C-peptide	-0.397	0.116	11.80	0.001*	0.672	0.536, 0.843
IL-6	-0.228	0.057	15.99	<0.001*	0.796	0.712, 0.890
Constant	-10.158	4.238	-	0.017*	-	-

## Discussion

This study included 88 pregnant women matched for age and gestational age. The present analysis aims to specifically examine the association between the insulin resistance markers (chemerin, visfatin, and C-peptide) and immunological markers (IL-6, IL-10, and IL-13) with GDM. In this cohort, a higher prevalence of GDM was observed among urban participants, as in our earlier study of the same cohort. We found that fasting glucose was elevated in GDM, whereas C-peptide levels were low [17]. Fasting glucose showed positive correlations with gestational age and was inversely associated with C-peptide. A systematic review and meta-analysis also showed that higher glucose levels were associated with gestational age and pregnancy outcomes, supporting our finding [18]. C-peptide reflects endogenous insulin secretion and β-cell activity. In early metabolic stress, compensatory hyperinsulinemia may maintain normoglycemia, whereas the failure of compensation may lead to glucose intolerance. The inverse association between C-peptide and glucose in our study may indicate the dysfunction of β-cell function secondary to glucotoxicity in women with GDM [4,17,18].

Chemerin, as an independent predictor of GDM, is likely to be associated with disease pathogenesis by worsening insulin resistance through adipose tissue dysfunction and inflammatory activation, further intensifying the physiological metabolic stress of pregnancy. Several studies in pregnant populations have reported elevated chemerin levels in women with GDM [14,19,20]. Our findings are consistent with these observations, as chemerin was positively associated with fasting glucose and C-peptide. However, various studies have suggested that BMI may influence chemerin levels. In this study, BMI was not included, which limits the generalizability of the observed increase in chemerin levels to GDM alone.

The IL-6 was inversely associated with fasting glucose and C-peptide in our research. The relationship between IL-6 and GDM has been inconsistent across studies. IL-6 has both proinflammatory and anti-inflammatory properties and is elevated in insulin-resistant states in various studies. The pregnancy is characterized by dynamic immunological adaptations; some reports have shown higher IL-6 levels in GDM, whereas others have not found consistent associations [9,21,22]. Our observation of an inverse association highlights the complexity of immune signalling during pregnancy, and in addition, cytokine measurements are subject to biological variability, and we measured only second-trimester levels in our study, which may not capture the whole dynamic changes.

IL-13 showed a positive association with C-peptide and visfatin, suggesting a possible interaction among immune mediators, adipokines, and insulin secretion in regulating glucose metabolism during pregnancy [13,23-26]. However, levels of IL-13 and visfatin did not differ between the study groups, which was found in our previous study. We did not find an association between chemerin, IL-6, IL-10, and visfatin.

In the multiple linear regression analysis, the parameters were selected based on the significance levels reported in our previous work. IL-6, C-peptide, chemerin, and gestational age independently predicted fasting glucose levels. Chemerin and gestational age were associated with higher glucose levels, whereas IL-6 and C-peptide showed inverse associations. In logistic regression analysis performed after excluding plasma glucose to avoid diagnostic circularity, chemerin independently increased the odds of GDM, while higher IL-6 and C-peptide levels were associated with lower odds. These findings suggest a complex interaction between adipokines, inflammatory mediators, and β-cell function in GDM. Although gestational age was adjusted for in regression models, other important confounders may have influenced the results. Factors such as prepregnancy BMI, gestational weight gain, dietary patterns, physical activity, and socioeconomic status were not fully incorporated into the final models. Additionally, inflammatory markers can be affected by subclinical infections or transient inflammatory states, which were not systematically evaluated. The interrelationship between adipokines and cytokines may also complicate the interpretation of independent effects.

Strengths and limitations

A key strength of this study is its matched design, which reduces confounding by age and gestational age. The simultaneous assessment of metabolic and immunological markers provides a broader perspective on immune-metabolic interactions in GDM. Furthermore, excluding plasma glucose from the logistic regression model helped avoid diagnostic overlap and allowed the identification of glucose-independent predictors.

However, several limitations must be acknowledged. The sample size was small. Measurements were limited to the second trimester without longitudinal follow-up, and correction for multiple testing was not performed. The cross-sectional design precludes establishing causality or temporal relationships between biomarker alterations and GDM development. We acknowledge that the urban-rural distribution differed between the groups and may act as a potential confounder due to differences in lifestyle, diet, and healthcare access. BMI, gestational weight gain, diet, physical activity, socioeconomic factors, and direct indices of insulin resistance are important determinants of GDM. However, these variables were not available in our dataset and therefore could not be included in the regression models, which may result in residual confounding. Given that this is a single-center study, the findings may not be generalizable to other populations.

Implications and future directions

These findings support the concept that GDM involves a multifactorial interplay between adipokines, inflammatory mediators, and β-cell function rather than isolated hyperglycemia. Chemerin appears to be a potential biomarker of metabolic risk in pregnancy, whereas the role of IL-6 may be more complex and dependent on inflammation status.

Future longitudinal studies with larger and more diverse populations are needed to clarify temporal relationships and causal pathways. The early pregnancy assessment of these biomarkers may help identify women at risk before overt hyperglycemia develops. Integrating metabolic, inflammatory, and clinical risk factors could improve risk stratification and guide preventive strategies.

## Conclusions

In conclusion, this study highlights the interplay between adipokines, inflammatory mediators, and β-cell function in gestational diabetes mellitus. Chemerin emerged as an independent predictor of GDM and was positively associated with fasting glucose, suggesting its potential role as a biomarker of metabolic dysfunction during pregnancy. IL-6 and C-peptide showed inverse associations with glucose, while IL-13 was positively associated with C-peptide and visfatin, indicating complex immune-metabolic interactions. These findings support the concept that GDM is an immunometabolic disorder and warrant further investigation in larger longitudinal studies.

## Appendices

Table 5 shows the sensitivity and coefficient of variation of study parameters.

**Table 5:** Sensitivity and CV of study parameters The measurement was done in duplicates, and the average was taken as the desired value CV, coefficient of variation; ELISA, enzyme-linked immunosorbent assay

ELISA assay	Manufacturer	Catalogue number	Detection range	Sensitivity	Intra-assay CV values	Inter-assay CV values
IL-6	Abbkine Scientific Co., Ltd. (Wuhan, China)	KTE6017	3.13-200 pg/mL	2 pg/mL	CV ＜10%	CV ＜12%
IL-10	Abbkine Scientific Co., Ltd. (Wuhan, China)	KTE6019	2.35-150 pg/mL	1 pg/mL	CV ＜10%	CV ＜12%
IL-13	Abbkine Scientific Co., Ltd. (Wuhan, China)	KTE6012	7.8-500 pg/mL	4 pg/mL	CV ＜10%	CV ＜12%
Visfatin	Abbkine Scientific Co., Ltd. (Wuhan, China)	KTE62221	25-400 pg/mL	1.0 pg/mL	CV ＜ 9%	CV ＜11%
Chemerin	BT Lab (Shanghai, China)	E143Hu	10-3000 ng/L	4.99 ng/L	CV < 8%	CV < 10%
Fasting glucose	Beckman Coulter (Brea, CA)	OSR6121	10-800 mg/dL	0.04 mmol/L	CV < 0.70%	CV < 1.25%
C-peptide	BT Lab (Shanghai, China)	E0009Hu	0.1-40 ng/mL	0.053 ng/mL	CV < 8%	CV < 10%

## References

[1] Modzelewski R, Stefanowicz-Rutkowska MM, Matuszewski W, Bandurska-Stankiewicz EM (2022). Gestational diabetes mellitus-recent literature review. J Clin Med.

[2] HAPO Study Cooperative Research Group (2009). Hyperglycemia and Adverse Pregnancy Outcome (HAPO) study: associations with neonatal anthropometrics. Diabetes.

[3] (2026). Diabetes atlas. https://diabetesatlas.org/resources/previous-editions/.

[4] Plows JF, Stanley JL, Baker PN, Reynolds CM, Vickers MH (2018). The pathophysiology of gestational diabetes mellitus. Int J Mol Sci.

[5] Ray GW, Zeng Q, Kusi P (2024). Genetic and inflammatory factors underlying gestational diabetes mellitus: a review. Front Endocrinol (Lausanne).

[6] Khambule L, George JA (2019). The role of inflammation in the development of GDM and the use of markers of inflammation in GDM screening. Adv Exp Med Biol.

[7] Krzyzanowska K, Krugluger W, Mittermayer F, Rahman R, Haider D, Shnawa N, Schernthaner G (2006). Increased visfatin concentrations in women with gestational diabetes mellitus. Clin Sci (Lond).

[8] Mohan V, Deepa M, Tandon N (2025). Prevalence of gestational diabetes mellitus in India: the ICMR-INDIAB national study (ICMR-INDIAB-24). Indian J Med Res.

[9] Valencia-Ortega J, González-Reynoso R, Ramos-Martínez EG, Ferreira-Hermosillo A, Peña-Cano MI, Morales-Ávila E, Saucedo R (2022). New insights into adipokines in gestational diabetes mellitus. Int J Mol Sci.

[10] Amirian A, Mahani MB, Abdi F (2020). Role of interleukin-6 (IL-6) in predicting gestational diabetes mellitus. Obstet Gynecol Sci.

[11] Ni J, Wang Y, Zhu J, Zheng T, Yu J, Xiong Y (2022). Interleukin-10 levels and risk of gestational diabetes mellitus: a systematic review and meta-analysis. J Matern Fetal Neonatal Med.

[12] Gong Z, Yi H, Zhang J (2025). Role of Arg1+ ILC2s and ILCregs in gestational diabetes progression. Sci Rep.

[13] Jiang YK, Deng HY, Qiao ZY, Gong FX (2021). Visfatin level and gestational diabetes mellitus: a systematic review and meta-analysis. Arch Physiol Biochem.

[14] Aldharee H, Makki YR, Hamdan HZ (2025). The association between chemerin levels and gestational diabetes mellitus: an updated systematic review and meta-analysis. Int J Mol Sci.

[15] Siddiqui S, Waghdhare S, Jha S, Dubey S (2019). Role of immunological markers in gestational diabetes mellitus-a brief review. Diabetes Metab Syndr.

[16] Metzger BE, Gabbe SG, Persson B (2010). International association of diabetes and pregnancy study groups recommendations on the diagnosis and classification of hyperglycemia in pregnancy. Diabetes Care.

[17] Bernea EG, Uyy E, Mihai DA (2022). New born macrosomia in gestational diabetes mellitus. Exp Ther Med.

[18] Zhao D, Liu D, Shi W (2023). Association between maternal blood glucose levels during pregnancy and birth outcomes: a birth cohort study. Int J Environ Res Public Health.

[19] Ma Z, Chu L, Zhang Y, Lu F, Zhu Y, Wu F, Zhang Z (2023). Is chemerin associated with gestational diabetes mellitus? A case-control study. Diabetes Metab Syndr Obes.

[20] Zhou Z, Chen H, Ju H, Sun M (2018). Circulating chemerin levels and gestational diabetes mellitus: a systematic review and meta-analysis. Lipids Health Dis.

[21] Gelen V, Şengül E, Atila G, Uslu H, Makav M (2017). Association of gestational diabetes and proinflammatory cytokines (IL-6, TNF-α and IL-1β). J Embryo.

[22] Srivastava N, Singh K, Singh N, Mahdi AA (2023). Association between serum interleukin-6, leptin and insulin in gestational diabetes mellitus - a cross- sectional study. J Diabetes Metab Disord.

[23] Sifnaios E, Mastorakos G, Psarra K (2019). Gestational diabetes and T-cell (Th1/Th2/Th17/Treg) immune profile. In Vivo.

[24] Milan KL, Shree RA, Nandana N, Leela R, Ramkumar KM (2025). Role of macrophages reprogramming in pathogenesis of gestational diabetes mellitus. Cytokine.

[25] Telejko B, Kuzmicki M, Zonenberg A (2009). Visfatin in gestational diabetes: serum level and mRNA expression in fat and placental tissue. Diabetes Res Clin Pract.

[26] Liang Z, Wu Y, Xu J, Fang Q, Chen D (2016). Correlations of serum visfatin and metabolisms of glucose and lipid in women with gestational diabetes mellitus. J Diabetes Investig.

